# Effects of treadmill exercise on the regulatory mechanisms of mitochondrial dynamics and oxidative stress in the brains of high-fat diet fed rats

**DOI:** 10.20463/jenb.2019.0005

**Published:** 2019-03-31

**Authors:** Jung-Hoon Koo, Eun-Bum Kang

**Affiliations:** 1 Exercise Biochemistry Laboratory, Korea National Sport University, Seoul Republic of Korea; 2 Division of Sports Science, Daejeon University, Daejeon Republic of Korea

**Keywords:** high fat diet, treadmill exercise, mitochondrial dynamics, oxidative stress

## Abstract

**[Purpose]:**

The purpose of this study was to investigate the effects of treadmill exercise on oxidative stress in the hippocampal tissue and mitochondrial dynamic-related proteins in rats fed a long-term high-fat diet (HFD).

**[Methods]:**

Obesity was induced in experimental animals using high fat feed, and the experimental groups were divided into a normal diet-control (ND-CON; n=12), a high fat diet-control (HFD-CON; n=12) and a high fat diet-treadmill exercise (HFD-TE; n=12) group. The rats were subsequently subjected to treadmill exercise (progressively increasing load intensity) for 8 weeks (5 min at 8 m/min, then 5 min at 11 m/min, and finally 20 min at 14 m/min). We assessed weight, triglyceride (TG) concentration, total cholesterol (TC), area under the curve, homeostatic model assessment of insulin resistance, and AVF/body weight. Western blotting was used to examine expression of proteins related to oxidative stress and mitochondrial dynamics, and immunohistochemistry was performed to examine the immunoreactivity of gp91phox.

**[Results]:**

Treadmill exercise effectively improved the oxidative stress in the hippocampal tissue, expression of mitochondrial dynamic-related proteins, and activation of NADPH oxidase (gp91phox) and induced weight, blood profile, and abdominal fat loss.

**[Conclusion]:**

Twenty weeks of high fat diet induced obesity, which was shown to inhibit normal mitochondria fusion and fission functions in hippocampal tissues. However, treadmill exercise was shown to have positive effects on these pathophysiological phenomena. Therefore, treadmill exercise should be considered during prevention and treatment of obesity-induced metabolic diseases.

## INTRODUCTION

Obesity is defined as a low-grade systemic inflammatory state, and it is known to induce various physiological changes such as inflammation, oxidative stress, mitochondrial dysfunction, and apoptosis^1^^-^^3^.

In energy metabolism, reactive oxygen species (ROS) are inevitably generated, but the hyperglycemia or insulin resistance induced by obesity leads to continuous production of ROS, which is a major cause of increased oxidative stress. The accumulation of ROS within mitochondria is generally known to be reduced by antioxidant enzymes such as catalase and superoxide dismutase (SOD) as part of homeostasis maintenance^4^. Nonetheless, the continuous production of ROS due to obesity increases oxidative stress in mitochondria and causes mitochondrial dysfunction. Notably, the oxidative stress generated in the mitochondria is produced by NADPH oxidase (NOX), a membrane bound enzyme complex. NOX2, an isoform of NOX, in particular, has been shown to play a key role in metabolic and neurodegenerative disorders^5^. NOX2 expression has been reported in various cell types including neurons and endothelial cells, and its largest expression is known to occur in microglial cells involved in immune/inflammatory reactions^6^^,^^7^.

Mitochondria are vital intracellular organelles responsible for biological oxidation in most eukaryotic cells. As cells forms constantly change, mitochondrial dynamics are regulated through repeated fusion (where the length of mitochondria increases) and fission (where mitochondria split into two)^8^^,^^9^. The maintenance of homeostasis by regulating changes in mitochondrial form is very important in maintaining normal mitochondrial function and requires control of both the quantity and quality of proteins associated with mitochondrial dynamics^10^. Several studies have identified the key proteins regulating the shift in mitochondrial form. Dynamin-related protein 1 (Drp1), mitochondrial fission 1 (Fis1), mitochondrial fission factor (MFF), mitochondrial dynamics 49 (MiD49), and MiD51 are known to be involved in facilitating mitochondrial fission, while mitofusin 1 (Mfn1), mitofusin 2 (Mfn2), and optic atrophy protein 1 (OPA1) are involved in mitochondrial fusion^8^^,^^11^.

Since mitochondria play a pivotal role in intracellular energy metabolism, its form and function are regulated through continuous cycles of fusion and fission^12^. A recent study reported that, an imbalance in mitochondrial dynamics may be associated with imbalances in energy metabolism such as diabetes and obesity^13^. This suggests the possibility that mitochondrial dysfunction might play a role in the pathogenesis of several metabolic disorders^14^^–^^16^. In other words, unbalanced mitochondrial dynamics in the tissues of the pancreas, liver, and muscle, caused by obesity, may increase the production of ROS that induces oxidative stress, which inhibits insulin signaling pathways and ultimately increases insulin resistance leading to diabetes^17^^-^^19^. Moreover, unbalanced mitochondrial dynamics have been observed in neurodegenerative disorders such as Alzheimer’s and Parkinson’s disease, where it was reported to aggravate conditions^20^^,^^21^. In other words, the slowdown in mitochondrial dynamics induced by obesity was shown to contribute to the pathogenesis of various metabolic disorders, and from this, it can be seen that solving the problem of obesity is the key for recovering normal mitochondrial dynamics.

Physical activity has recently been recognized as an effective way to enhance mitochondrial function, and exercise has been reported to improve damaged mitochondrial dynamics in various disorders. Fealy et al.^22^, in particular, reported alleviation of insulin resistance when exercise was used to partially restore the increase in fission and decrease in fusion of mitochondria in the human skeletal muscle where insulin resistance occurs. Treadmill exercise has also been reported to have a positive influence on recovering cognitive function by restoring the balance of mitochondrial dynamics based on an increase in Drp1, a protein involved in mitochondrial fission present in neurodegenerative disorders^23^^,^^24^. Nonetheless, contrasting results have also been reported, where exercise led to increased or decreased expression of proteins related to mitochondrial fission or fusion^25^^,^^26^. This may be attributed to the changes caused by exercise intensity, disease pathology, tissue type, and various experimental conditions. In particular, previous studies that examined the effects of exercise on unbalanced mitochondrial dynamics have mostly focused on skeletal muscles, while there is a lack of studies on brain tissues. Notably, brain hippocampal tissue plays a central role in human cognition such as learning and memory and has a deep connection with neurodegenerative disorders such as Alzheimer's, which supports the significance of studies on the hippocampus.

As previously mentioned, reducing obesity through exercise has been reported to have a positive effect on energy metabolism, and numerous studies have investigated the role of exercise in improving pathological physiology in peripheral tissues. However, there are currently an extremely limited number of studies on the role of exercise and obesity in nerve cell mitochondrial dynamics. Thus, this study aims to examine the changes in the factors related to nerve cell mitochondrial dynamics in the brain, when long-term, high-fat diet obesity-induced lab animals perform treadmill exercise.

## METHODS

### Experimental animals

This study was approved by the Institutional Animal Care and Use Committee at H University prior to performing the experiments (KNSU-IACUC-2017-01). The lab animals were 8-week old male Sprague Dawley rats purchased from KOATECH, Co., Ltd and reared until 24 weeks in an animal lab at H University (temperature 22±2 ℃, humidity 50±5 %, and a 12 h light-dark cycle). When the rats reached 24 weeks of age, obesity was induced in some rats (n = 24) with high-fat feed (D12492; carbohydrate: 20 %, fat: 60 %, protein: 20 %) purchased from the Central Lab, Animal Inc., for 20 weeks, while they were allowed liberal intake and supply of water. The lab animals were categorized into three groups: normal diet-control (ND-Con, n=12), high fat diet-control (HFD-Con, n=12), and high fat diet-treadmill exercise (HFD-TE, n=12).

### Treadmill exercise

The HFD-TE group was acclimatized with pre-training for 30 min a day for five days, on a rodent treadmill (DJ2-242, Daejong Instrument Industry Co. Seoul, Korea) fixed at a 0 % gradient. After pre-training was complete, the main exercise was carried out five days a week for eight weeks. Fixing the gradient at 0 %, the animals were given the Maximal Graded Moderate to Intensive Exercise Program suggested in Kang et al. (Initial 5 min at 8 m/min, then 5 min at 11 m/min, and finally 20 min at 14 m/min)^27^.

### Oral glucose tolerance test (OGTT)

Twelve hours following the 8-week treadmill exercise, plasma glucose was measured from the tail of the rats in fasting state. Next, 1 mL triple distilled water and 0.3 mL glucose were mixed to prepare a 30 % glucose concentrate, which was administered to the rats at 1 mL∙kg-1. Blood was then taken from the rat tail five times (0 min, 30 min, 60 min, 90 min, and 120 min) and using a blood glucose monitoring device (Gluco-CardⅡ, Daichi Kagaku. Co., Kyoto, Japan), plasma glucose was measured and the reaction area for total glucose secretion (area under the curve; AUC0-120, mg/dl-1·min-1) was determined.

### Tissue samples collection

Twenty four hours after the 8-week’s treadmill exercise, the rats were anesthetized by intraperitoneal injection of Rompun/Zoletil mixture (2:1, 10 mg/kg), and to estimate protein expression, brain tissue, blood, and abdominal adipose tissue were removed from seven rats. The extracted brain tissue was separated into the cerebral cortex and hippocampus, which were rapidly cooled in liquid nitrogen and stored in a -80 ℃. After collection, blood from the heart was centrifuged to obtain serum for analyzing glucose and insulin. The abdominal adipose tissue was removed by incision and washed with cold physiological saline, and after removing the moisture with gauze, was weighed. For immunohistochemical staining, five rats were anesthetized, their thoracic cavities were opened, and 50 mM phosphate-buffered saline was injected through the left ventricle for 10 min, and perfused with 4 % paraformaldehyde (PFA) fixative dissolved in 100 mM phosphate buffer. After perfusion, the brain was removed and placed in 4 % PFA for 4-h fixation at 4 ℃. The fixed brain tissue was left to settle for two days in 30 % sucrose solution. Rodent Brain Matrix (RBM-4000C, 1 mm coronal section, ASI Instruments, Inc, USA) was used to extract regions of the cortex and hippocampus (from –4.88 mm to –1.76 mm from bregma) from the fixed brain tissue (whole brain). A freezing microtome (Leica, Nussloch, Germany) was then used to prepare a 40 μm thick serial coronal section.

### Biochemical analysis 

The blood collected by cardiac perforation was centrifuged (FLETA-5 centrifuge, Hanil Biomed Inc. Korea) and the serum was collected. The glucose, insulin, triglyceride (TG), and total cholesterol (TC) concentrations were analyzed by Green Cross Corp. The HOMA-IR index was calculated from the concentrations of glucose and insulin according to the following equation: HOMA-IR = fasting serum glucose (mmol/L) × fasting serum insulin (μU/Ml)/22.5.

### Mitochondria isolation

The Mitochondria Extraction Kit (IMGENEX Corporation, San Diego, CA, USA) was used to isolate mitochondria. One mL homogenizing buffer was added per 100 mg of hippocampal tissue, followed by 10-min centrifugation at 4 °C and 3,000 rpm, the supernatant was removed and centrifuged again for 30 min at 4 °C at 12,000 rpm. The centrifuged supernatant (cytosolic fraction) was isolated as cytosol, while the remaining pellet was mixed thoroughly with 1 mL suspension buffer and centrifuged again for 10 min at 4 °C and 12,000 rpm. The supernatant was removed, the pellet was resuspended in 1 mL suspension buffer, and after another 10-min centrifugation at 4 °C and 12,000 rpm, the supernatant was removed and the resulting pellet was dissolved in 1 mL Complete Mitochondrial lysis buffer for 30 min at 4 °C. The separated mitochondrial extract was centrifuged for 5 min at 4 °C and 12,000 rpm, and the supernatant (mitochondria fraction) was collected.

### Western blot

Hippocampal tissue from isolated brain tissues was homogenized using lysis buffer and a homogenizer. It was then centrifuged for 15 min at 4 ℃ and 13,000 g, and the total protein content in the supernatant was quantified by the Bradford method. Protein (30 μg) was separated by electrophoresis in SDS-Polyacrylamide gel (7%, 10%), transferred to PVDF membrane (Amersham, Arlington Heights, IL, USA), blocked in 1× TBS-T solution containing 3 % BSA for 1 h at room temperature. The proteins and the corresponding primary antibody (Table 1) were left to react at 4 ℃ (for over 12 h). The following day, the membrane was washed three times in TBS-T buffer for 10 min, and the secondary antibody was reacted for 1 h at room temperature. After another three 10 min washes with TBS-T buffer, the membrane was added to the WBLR solution (Western Blotting Luminol Reagent SC-2048, Santacruz Biotechnology, USA) for 1-min coloration, and the resulting membrane was scanned using an image analysis system (Molecular Imager ChemiDoc XRS System, Bio-Rad, Hercules, CA, USA). Next, the Quantity One 1D Analysis Software (Bio-Rad, Hercules, CA, USA) was used to estimate the protein content

**Table 1:** Primary antibodies

Antibody	Source	Vender	Catalog No.
Mfn1	mouse monoclonal	Santa Cruz	sc-166644
Mfn2	mouse monoclonal	Santa Cruz	sc-100560
OPA1	mouse monoclonal	Santa Cruz	sc-393296
Drp1	mouse monoclonal	Santa Cruz	sc-271583
Fis1	mouse monoclonal	Santa Cruz	sc-376447
CS	mouse monoclonal	Santa Cruz	sc-390693
gp91 phox	rabbit polyclonal	Santa Cruz	sc-20782
SOD-2	mouse monoclonal	Santa Cruz	sc-133134
Catalase	mouse monoclonal	Santa Cruz	sc-271803
β-Actin	rabbit polyclonal	Santa Cruz	sc-47778

Mfn1; Mitofusin1; OPA; Optic atrophy 1; Drp1; Dynamin-related protein 1; Fis; Mitochondrial fission 1; CS; Citrate synthase; gp91 phox; Glycoprotein 91 phagosome oxidase; SOD-2; Superoxide dismutase-2.

### Immunofluorescence (IF)

For the five rats to be analyzed by immunofluorescence assay, the brain tissue samples from each group (5 rats) were washed three times for 10 min using 0.01 M PBS according to the free-floating method. Then, each sample was placed in a beaker containing 0.01 M sodium citrate for 60 min for incubation at 90 °C, followed by blocking with 10 % normal donkey serum for 60 min. After blocking, the primary antibody (gp91phox) was added and the sample was left overnight for 12 h at 4 ℃, then washed three times for 5 min using 0.01 M PBS. The sample was then reacted with the secondary antibody (Alexa-488 conjugated donkey anti-mouse; 1:200 dilution, Jackson Immunochemicals, West Grove, PA, USA) for 2 h and washed four times, and each sample was moved to a slide and sealed using mounting solution (Vector Laboratories, Burlingame, CA, USA). The slides were imaged using an immunofluorescence microscope (Leica Microsystems, TCS SP8, Germany).

### Data processing

All data obtained in this study were used to generate descriptive statistics (mean ± SEM) using the 18.0 SPSS program for Windows. Intergroup differences were analyzed by one way ANOVA, and when a significant intergroup difference was found, the Bonferroni method was used as the post hoc test. Here, the statistical level of significance was set as α=0.05.

## RESULTS

### Effects of TE on body weight in HFD-fed rats

**Figure 1:**
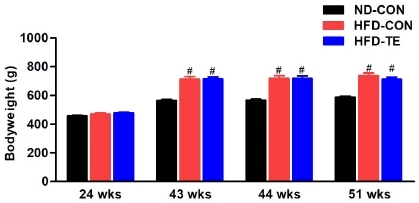
Effects of treadmill exercise (TE) on bodyweight in each group. 24 wks.: before high-fat fed, 43 wks.: after high-fat fed, 44 wks.: before TE, 51 wks.: after TE. Bonferroni post hoc test was used after ANOVA. Values are means ± SEM. #Denotes statistical difference from the ND-CON group (p < .05).

The changes in weight based on high-fat diet and treadmill exercise were measured at weeks 24, 43, 44, and 51, and the results are presented in Fig. 1. No significant difference was found between the three groups prior to the high-fat diet (Week 24), while significant intergroup differences in weight were found (F=32.969, p<0.05) after 20 weeks of high-fat diet (Week 43). The Post hoc test showed that compared to the ND-Con group, both the HFD-Con and HFD-TE groups had significant increases in weight (p<0.05). There was no difference between the HF-Con and HFD-TE groups.

### Effects of TE on TG, TC, AUC, HOMA-IR, and AVF/bodyweight in HFD-fed rats

The area under the curve of glucose (AUC), HOMA-IR, and AVF/bodyweight, obtained from testing the plasma lipids TG, TC, and OGTT, is given in Table 2. Significant differences in TG were found between the groups (F=31.654, p<0.05). The Post hoc test showed that, compared to ND-Con, TG was significantly higher in the HFD-Con (p<0.05) and HFD-TE (p<0.05) groups, while no significant difference was found between the two groups. There were also significant differences in TC between the groups (F=29.089, p<0.05). TC in the HFD-Con group was significantly higher than in the ND-Con (p<0.05) and HFD-TE (p<0.05) groups. The AUC showed significant intergroup differences (F=16.618, p<0.05). The post hoc test showed that TC was significantly higher in the HFD-Con than in the ND-Con (p<0.05) and HFD-TE (p<0.05) groups. Significant intergroup differences were found in AVF (F=90.614, p<0.05). The post hoc test showed that AVF in the HFD-Con group was significantly higher than in the ND-Con (p<0.05) and HFD-TE (p<0.05) groups.

**Table 2:** Comparison of TG, TC, AUC, HOMA-IR, and AVF/body weight

Metabolic parameters	ND-CON	HFD-CON	HFD-TE
TG (mg/dl)	97.00±2.59	130.00±2.43^#^	124.00±4.38^#^^*^
TC (mg/dl)	108.29±5.15	150.00±3.48^#^	120.71±2.94^#^^*^
AUC (mg/dl∙min)	43416.25±2004.77	57106.25±1045.13^#^	46520.00±2046.74^#^^*^
HOMA-IR	1.74±0.16	5.89±0.27^#^	3.80±0.34^#^^*^
AVF/body weight (%)	5.48±0.17	8.82±0.19^#^	6.17±0.06^#^^*^

Values are presented as means ± SE, ND-CON; Normal diet-control, HFD; High fat diet, TE; Treadmill exercise, TG; Triglyceride, TC; Total cholesterol, AUC; Area under the curve, HOMA-IR; Homeostasis model assessment-insulin resistance, AVF; Abdominal visceral fat. #Denotes statistical difference from the ND-CON group. *Denotes statistical difference from the HFD-CON group (p<0.05).

### Effect of TE on the expression of CS, gp91phox, SOD-2 and catalase in the hippocampus of HFD-fed rats

**Figure 2:**
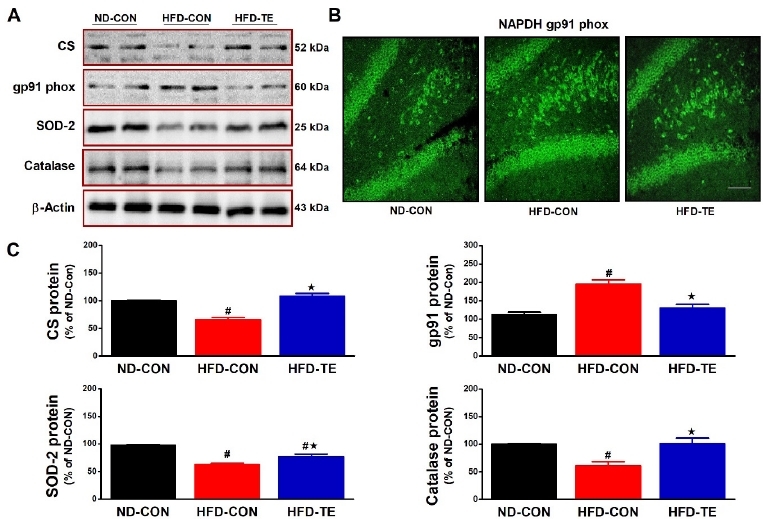
Effects of TE on the expression of CS, gp91phox, SOD-2 and catalase in the hippocampus of HFD-fed rats. (A) Representative western blots of CS, gp91phox, SOD-2 and catalase proteins. (B) Immunofluorescence images of NADPH gp91phox from each group are shown (n=5). (scale bar=100 μm). (C) Densitometric analysis of the western blot bands normalized to β-Actin. The data shown in the western blot are means from seven rat brains. β-Actin was probed as an internal control. Bonferroni post hoc test was used after ANOVA. Values are means ± SEM. #Denotes statistical difference from the ND-CON group. *Denotes statistical difference from the HFD-CON group (p < .05).

The effect of treadmill exercise on CS, gp91phox, SOD-2, and Catalase expression is presented in Fig. 2. Significant intergroup differences were found in CS expression (F=36.719, p<0.05). The post hoc test showed that CS expression was significantly lower in the HFD-Con group than in the ND-Con group (p<0.05) and that expression in the HFD-TE group was higher than in the HFD-Con group (p<0.05). Immunohistology found that compared to the ND-Con group gp91phox immunoreactivity was higher in the HFD-Con and lower in the HFD-TE group (Fig. 2B). There were significant intergroup differences in Gp91phox protein expression (F=21.297, p<0.05). In the post hoc test, compared to the ND-Con group, expression was significantly higher in the HFD-Con (p<0.05) and significantly lower in the HFD-TE (p<0.05) group. Significant intergroup differences were also found in SOD 2 expression (F=37.462, p<0.05), with the post hoc test showing that, compared to the ND-Con group, there was a significant decrease in expression in the HFD-Con group (p<0.05), and a significant increase in expression in the HFD-TE (p<0.05) group. There were also significant intergroup differences in catalase expression (F=11.116, p<0.05), with the post hoc test showing that, compared to the ND-Con group, there was a significant decrease in expression in the HFD-Con (p<0.05) and a significant increase in expression in the HFD-TE (p<0.05) group.

### Effects of TE on the expression of mitochondrial fusion-related proteins in the hippocampus of HFD-fed rats

**Figure 3:**
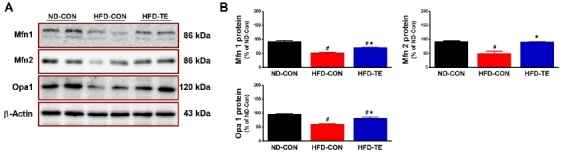
Effects of TE on the expression of mitochondrial fusion-related proteins in the hippocampi of HFD-fed rats. (A) Representative western blots of mitochondrial fusion-related proteins (Mfn1, Mfn2, Opa1). (B) Densitometric analysis of the western blot bands normalized to β-Actin. The data shown in the Western blot are means from seven rat brains. β-Actin was probed as an internal control. Bonferroni post hoc test was used after ANOVA. Values are means ± SEM. #Denotes statistical difference from the ND-CON group. *Denotes statistical difference from the HFD-CON group (p < .05).

The effect of exercise on mitochondrial fusion factors (Mfn1, Mfn2, and Opa1) is given in Fig. 3. When the mitochondrial fusion factors were analyzed using one-way ANOVA, significant intergroup differences were found in Mfn1 expression (F=53.067, p<0.05) with the post hoc test showing that expression in the HFD-Con group was significantly lower than in the ND-Con (p<0.05) or HFD-TE group (p <0.05). Significant differences were also found in Mfn2 expression (F=20.284, p<0.05), and the post hoc test showed that expression in the HFD-Con group was significantly less than in the ND-Con group (p<0.05) while expression in the HFD-TE group was significantly higher (p<0.05). Finally significant differences were found in Opa1 expression (F=27.285, p<0.05), with expression in the HFD-Con group significantly lower than in the ND-Con group (p<.05) while expression in the HFD-TE group was significantly higher (p<.05).

### Effects of TE on the expression of mitochondrial fission-related proteins in the hippocampus of HFD-fed rats

**Figure 4:**
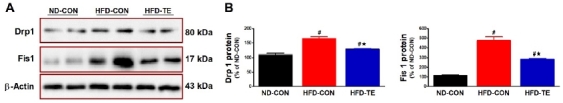
Effects of TE on the expression of mitochondrial fission-related proteins in the hippocampi of HFD-fed rats. (A) Representative western blots of mitochondrial fission-related proteins (Drp1, Fis1). (B) Densitometric analysis of the western blot bands normalized to β-Actin. The data shown in the western blot are means from seven rat brains. β-Actin was probed as an internal control. Bonferroni post hoc test was used after ANOVA. Values are means ± SEM. #Denotes statistical difference from the ND-CON group. *Denotes statistical difference from the HFD-CON group (p < .05).

The effect of treadmill exercise on mitochondrial fission factors (Drp1 and Fis1) is given in Fig. 4. Analyzing mitochondrial fission factors using one-way ANOVA found significant intergroup differences for Mfn1 expression (F=32.315, p<0.05), with the post hoc test finding a significantly higher expression in the HFD-Con group than the ND-Con group (p<0.05) while expression was significantly lower in the HFD-TE group (p<0.05). Significant differences were also found for Fis1 (F=55.883, p<0.05), and the post hoc test showed significantly higher expression in the HFD-Con group than in the ND-Con group (p<0.05) while HFD-TE expression in the HFD-Con group was significantly lower (p<0.05).

## DISCUSSION

This study investigated the effects of treadmill exercise on oxidative stress and the fusion and fission of mitochondria in the hippocampal tissue of lab animals, where obesity was induced by a long-term, high-fat diet. The 20-week high-fat diet increased the weight and abdominal visceral fat in the animals while decreasing the level of HOMA-IR, an indicator of insulin resistance, and their ability to control plasma glucose (AUC from OGTT data). It also increased the serum concentrations of TG and TC. However, treadmill exercise was shown to improve these pathological symptoms associated with obesity. The long-term, high-fat diet was also shown to promote the fusion of mitochondria in the hippocampal tissue while reducing their fission, thereby partially disrupting mitochondrial dynamics. Moreover, the high-fat diet led to an increase in oxidative stress due to increased activity of NADPH gp91phox and decreased expression of CS, an indicator of mitochondrial energy metabolism activity. However, treadmill exercise suppressed the oxidative stress by enhancing the expression of antioxidant enzymes, and this improvement alleviated the imbalance in mitochondrial dynamics. A detailed discussion is given below.

The long-term, high-fat diet increased the weight, abdominal visceral fat, and serum TG and TC concentrations in the lab animals. The level of HOMA-IR and its AUC also increased. While conditions like impaired glucose tolerance, hyperlipidemia, or type II diabetes cannot be conclusively demonstrated, their negative influence on energy metabolism can be seen, such as the decrease in the ability to control plasma glucose or the increased incidence of hyperinsulinemia compared to that seen in rats fed a normal diet. However, treadmill exercise reduced the increase in weight due to high-fat diet and improved AUC, HOMA-IR, and abdominal fat content while reducing the concentrations of plasma TG and TC. These results are in concordance with previous studies that reported the effect of exercise on improving the negative influence of obesity on energy metabolism^28^^,^^29^. In other words, treadmill exercise may contribute to increasing the utilization of glucose and triglycerides and enhance insulin sensitivity, which suggests that it may be an effective intervention for improving insulin resistance and the ability to control plasma glucose^30^.

According to a recent study, oxidative stress plays a critical role in linking obesity to cognitive dysfunction and brain function is sensitive to oxidative stress, which was reported to increase in obesity^31^. This study analyzed gp91phox, a membrane subunit of the NOX isoform NOX2. All groups that were fed a long-term, high-fat diet showed increased activation of gp91phox in the hippocampus, compared to the normal diet group. Immunohistochemical assays also found increased immune reaction from gp91phox in the hippocampus (DG). Nevertheless, treadmill exercise downregulated the activation of gp91phox; i.e., treadmill exercise effectively inhibited the activation of NADPH oxidase, which can produce ROS. This finding agreed with the result of a previous study that reported an increase in NOX activation due to a high-fat diet although the analyzed tissue (aorta) was different^32^. It was also consistent with NOX activity being downregulated by treadmill exercise in the aorta of a high-fat diet-induced obesity model^33^. In addition, expression of catalase and SOD-2, the mitochondrial antioxidant enzymes that protect the cell from oxidative stress, was reduced in both the HD-Con and ND-Con groups, which was increased by treadmill exercise. Likewise, expression of citrate synthase, an enzyme that is part of the Krebs cycle in mitochondria and is an indicator of oxidative capacity, was reduced in the high-fat diet group, while treadmill exercise led to increased expression. The antioxidant enzymes SOD-2 and catalase are the major defense against ROS generated during exercise, so their expression increases as a response to exercise^34^^,^^35^. Treadmill exercise in this study also led to increased SOD-2 and catalase activities, and notably, the increased activity of citrate synthase, an enzyme specialized for mitochondrial substrates and the most important enzyme in the Krebs cycle, confirmed the improvement of mitochondrial dysfunction.

Mitochondrial dynamics are a crucial mechanism in the maintenance of mitochondrial homeostasis, and oxidative stress was reported to increase due to unbalanced mitochondrial dynamics in obesity. This study also showed that, in the HFD-Con group, where obesity was induced, the expression of proteins related to mitochondrial fission, Drp1 and Fis1, increased, whereas the expression of proteins related to mitochondrial fusion, Mfn1, Mfn2, and Opa1, decreased. This can be explained by an excessive increase in mitochondrial fusion partially reducing mitochondrial function. Obesity causes an imbalance in mitochondrial fusion and fission, which reduces the mitochondria content. It is also associated with defects in neuronal development, plasticity, and function^36^. In particular, Mfn2 inhibition is related to substrate oxidation of the ETC complex, cellular metabolism, and a decrease in membrane potential in obesity^37^. Despite being focused on skeletal muscle, a study on a long-term high-fat diet reported decreased levels of Mfn1 and Mfn2 protein and increased levels of Fis1 and Drp1 protein^38^. On the other hand, treadmill exercise was shown to decrease the expression of mitochondrial fission proteins, Drp1 and Fis1, while increasing the expression of mitochondrial fusion proteins, Mfn1, Mfn2, and Opa1. Although a direct comparison is difficult due to the lack of studies on the link between obesity and mitochondrial dynamics in the brain, Axelrod et al.^39^ reported that exercise may control mitochondrial fusion and fission in human muscle and thus induce post-translational modifications in mitochondria, and Konopka et al.^40^ reported that 12 weeks of aerobic exercise training led to a similar increase in the expressions of MFN1 and MFN2 in skeletal muscle of both the young and elderly subjects. Zampieri et al.^41^ reported increased expression of OPA1 upon 12-week electrical stimulation of skeletal muscle. Considering that these studies examined mitochondrial dynamics in skeletal muscle cells, exercise can be seen to be an effective way to improve the imbalance in mitochondrial dynamics in the brain.

To conclude, a long-term high-fat diet induced increased weight in lab animals, a decrease in the ability to control plasma glucose, and hematopathological changes that elevate the oxidative stress in the mitochondria of the brain hippocampal tissue to cause an imbalance in mitochondrial dynamics. However, treadmill exercise improved the pathological changes induced by obesity, so that it may contribute to functional improvement in mitochondria in the hippocampal tissue. Thus, this study suggests the need to consider treadmill exercise in the prevention and treatment of mitochondrial dysfunction in the brain hippocampal tissue and in the alleviation of the pathological changes induced by obesity.
